# Membranous Extracellular Matrix-Based Scaffolds for Skin Wound Healing

**DOI:** 10.3390/pharmaceutics13111796

**Published:** 2021-10-27

**Authors:** Lin-Cui Da, Yi-Zhou Huang, Hui-Qi Xie, Bei-Hong Zheng, Yong-Can Huang, Sheng-Rong Du

**Affiliations:** 1 Center of Reproductive Medicine, Fujian Maternity and Child Health Hospital, Affiliated Hospital of Fujian Medical University, Fuzhou 350001, China; dalincui@163.com (L.-C.D.); zhengbeihong2010@163.com (B.-H.Z.); 2 Laboratory of Stem Cell and Tissue Engineering, Orthopedic Research Institute, State Key Laboratory of Biotherapy and Cancer Center, West China Hospital, Sichuan University and Collaborative Innovation Center of Biotherapy, Chengdu 610041, China; huangyizhou@wchscu.cn; 3 Shenzhen Engineering Laboratory of Orthopaedic Regenerative Technologies, Department of Spine Surgery, Peking University Shenzhen Hospital, Shenzhen 518036, China; y.c.huang@connect.hku.hk

**Keywords:** chronic wounds, extracellular matrix, membranous scaffolds, skin substitutes, wound healing

## Abstract

Membranous extracellular matrix (ECM)-based scaffolds are one of the most promising biomaterials for skin wound healing, some of which, such as acellular dermal matrix, small intestinal submucosa, and amniotic membrane, have been clinically applied to treat chronic wounds with acceptable outcomes. Nevertheless, the wide clinical applications are always hindered by the poor mechanical properties, the uncontrollable degradation, and other factors after implantation. To highlight the feasible strategies to overcome the limitations, in this review, we first outline the current clinical use of traditional membranous ECM scaffolds for skin wound healing and briefly introduce the possible repair mechanisms; then, we discuss their potential limitations and further summarize recent advances in the scaffold modification and fabrication technologies that have been applied to engineer new ECM-based membranes. With the development of scaffold modification approaches, nanotechnology and material manufacturing techniques, various types of advanced ECM-based membranes have been reported in the literature. Importantly, they possess much better properties for skin wound healing, and would become promising candidates for future clinical translation.

## 1. Introduction

Chronic nonhealing wounds, including diabetic ulcers, pressure ulcers, venous ulcers and arterial ulcers, remain a major medical problem that poses a heavy burden on the patients, their families, and the healthcare system [1,2]. The situation may become worse because of a growing population of the elderly and an increasing morbidity of patients [3]. Undoubtedly, there is an urgent need for effective biomaterials to repair wounds in a shorter period of time, to improve the functional restoration of injured skin, and to reduce scar tissue formation. 

Body membranes are thin layers of cells or tissues covering the surface of body, the internal organs, or the body cavities. Generally, they can be classified into two categories: (1) the epithelial membranes, and (2) the connective tissue membranes [4]. The epithelial membranes, which are composed of epithelial tissue and fibrous connective tissue, can be further divided into (1) the cutaneous membrane (i.e., skin), (2) the serous membranes, such as pleura, peritoneum, and amniotic membrane, and (3) the mucous membranes [4]. Unlike epithelial membranes, connective tissue membranes (e.g., periosteum, fascia, and synovial membrane) are typically composed of cells, ground substance, and connective tissue fibers [4].

After decellularization, extracellular matrix (ECM) scaffolds obtained from different types of body membranes retain a variety of bioactive substances such as the growth factors, collagen, laminin, fibronectin, and polysaccharide. Notably, they possess ultrastructure features similar to that of the natural tissues [5,6,7]. These microporous thin films have a distinct advantage in the mass exchange between tissues [8,9]. Particularly, scaffolds in a thin planar form are favorable for high density cell seeding and the migration of repair cells from adjacent tissues [8,10]. These merits make membranous ECM scaffolds extremely attractive for skin wound healing. 

Indeed, the safety and efficiency of several membranous ECM scaffolds have been verified in many clinical practices; however, some of their physicochemical properties, such as the mechanical strength and the degradation characteristics, are far from satisfactory for broad applications. This is partially due to the damage of the crosslinked networks of natural tissues during scaffold preparation, especially the use of acids, alkalis or proteases for decellularization. It is well-known that an ideal scaffold for tissue repair should possess good biocompatibility, robust bioactivity, suitable degradation, and proper mechanical properties. Therefore, attempts to endow traditional membranous ECM scaffolds with desired properties have been the focus of many researches. For instance, to meliorate the physical or chemical defects of traditional ECM scaffolds, different crosslinking methods have been developed [11]. Furthermore, diverse macromolecules, either natural or synthetic, have been used as functional additives to produce ECM-based implants [12,13,14,15,16]. In light of the functional requirements of normal wound healing, many types of biomolecules, nanoparticles, and drugs have been utilized to engineer new generations of ECM-based biomaterials, which can stimulate a specific wound healing stage or event to facilitate chronic wound healing. 

In this article, we aim to review the development of membranous ECM-based scaffolds for skin wound healing. After a brief introduction of the traditional scaffolds, recent advances in the scaffold modification and the fabrication of new ECM-based membranes are summarized. Future research directions and perspectives on the scaffolding strategies are provided.

## 2. Traditional ECM Scaffolds Derived from Body Membranes for Skin Wound Healing

**Figure 1:**
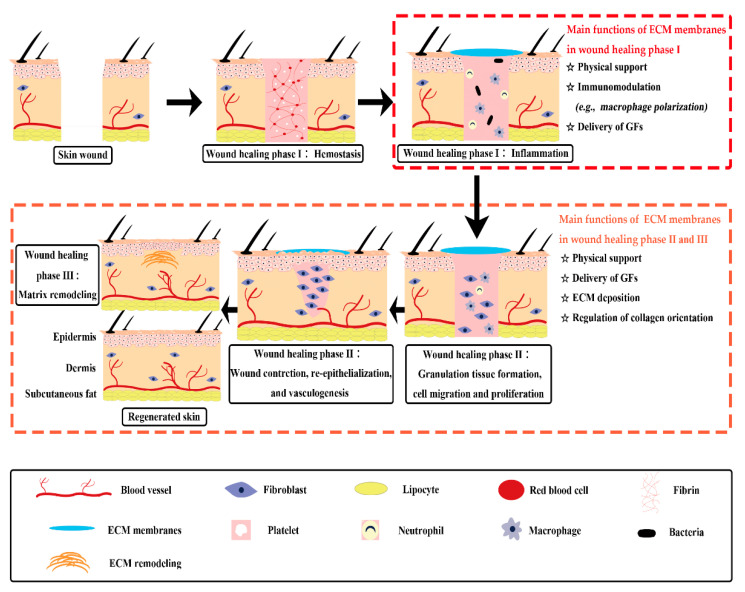
Diagram depicting concept and mechanisms of skin wound healing assisted by traditional ECM membranes. ECM membranes have multiple functions in the different phases of cutaneous wound healing. ECM: extracellular matrix; GFs: growth factors.

Traditional ECM membranes derived from human or animal tissues, such as pericardium, peritoneum, and chorion, have been utilized to facilitate skin wound healing [17,18,19,20,21,22]. Among them, acellular dermal matrix (ADM), small intestinal submucosa (SIS), and acellular amniotic membrane (AAM) are representative biomaterials that have been commercialized and extensively applied in the clinic [21,23,24]. The exact wound repair mechanism of membranous ECM scaffolds in living organisms remains to be fully understood. But it has been assessed that, besides physical support, traditional ECM membranes have functions of immunomodulation, growth factor stimulation and ECM regulation, which can trigger several crucial events during wound healing process (Figure 1) [10]. Chronic wounds usually experience a prolonged inflammation phase with some abnormal healing events. Some ECM membranes, like SIS and acellular pericardium, were proved to have immunomodulatory ability. They are capable of triggering the macrophages to express a predominant M2-like phenotype, which can secret pro-healing cytokines to initiate the anti-inflammatory and pro-remodeling process [25,26]. Moreover, some ECM components possess bioactive motifs to regulate cell adhesion and proliferation, such as the Arg–Gly–Asp (RGD) motif. The special domain of RGD peptide is capable of converting the inflammatory response towards a pro-healing response through the binding with integrins of macrophages to modulate signaling pathways involved in cell migration, adhesion, and inflammatory activation [27]. Besides, the inherent growth factors of ECM membranes may provide a complex signaling milieu to stimulate granulation tissue formation, moderate cell transition, angiogenesis, and matrix formation and remodeling during the wound healing phases [28,29].

### 2.1. Acellular Dermal Matrix (ADM)

Produced from human or animal skin, ADM is favorable for full-thickness skin wound healing and can reduce scar tissue formation [21,30,31,32]. After transplantation in the wound bed, ADM enhances the synthesis of hyaluronic acid and induces wound angiogenesis [29,33,34]. Currently, there are several ADM products from human skin, such as the AlloDermTM, GraftJacket^®^, and SureDerm^®^ [35]. AlloDerm™ has been utilized to cover deep burn wounds in a case series. The application of AlloDerm™ resulted in excellent graft take, good elasticity, little contracture, and few scarring [36]. GraftJacket^®^ has been recommended in the treatment of diabetic wounds or the replacement of damaged or inadequate integumental tissue [37]. When compared with standard wound care, it was reported that a single application of GraftJacket^®^ can reduce the mean wound healing time of diabetic foot ulcers [38]. 

Comparing with ADM derived from human skin, animal ADM products are more cost-effective and more frequently applied for large skin defects [21]. Some animal ADM, such as those from bovine, porcine, and fish skin, have been approved by the US Food and Drug Administration [39,40,41]. For instance, Kerecis™ graft, a newly-approved ADM product from fish skin, is very attractive for wound management because of the anti-inflammatory property of its exclusive omega-3 polyunsaturated fatty acids [42]. Further, the Kerecis™ graft avoids the risk of potential viral and prion transmission, which might be seen in mammalian-derived products [34]. According to recent clinical studies, the Kerecis™ graft can heal acute or chronic deep skin wounds with a shorter healing time than conventional wound treatment [41,42].

### 2.2. Small Intestinal Submucosa (SIS)

SIS is a membranous ECM scaffold derived from porcine small intestine. It has attracted considerable attention in clinical applications for tissue regeneration, mainly because of the good capabilities of activating immune mediators, inducing angiogenesis, and promoting reepithelialization [7,43,44]. These effects are likely due to the release of growth factors, such as basic fibroblastic growth factor, transforming growth factor-β (TGF-β), and vascular endothelial growth factor (VEGF) [45,46]. Mechanistically, SIS can orchestrate wound remodeling by eliciting a response of macrophages towards a M2 phenotype rather than a M1 phenotype, where the M1 phenotype can lead to prolonged inflammation and scarring [47,48]. Furthermore, SIS has a unique effect on the inhibition of matrix metalloproteinases [49]. Based on these merits, SIS is applicable for treating chronic wounds, where the microenvironment is harsh and the matrix metalloproteinases are abundant [46,50,51]. 

Taking Oasis^®^ Wound Matrix as an example, the clinical safety and efficiency of this SIS product have been confirmed by a clinical trial, in which 130 chronic leg ulcer cases were involved [52]. In this study, Oasis^®^ Wound Matrix demonstrated 40% of complete healing at 12 week after treatment, while the standard care group just resulted in 29% of complete healing [52]. In another study, chronic venous ulcers treated with SIS Wound Matrix resulted in a significant decrease in the expression of matrix metalloproteinases and pro-inflammatory cytokines, while the level of TGF-β was significantly increased [53]. These results revealed that SIS Wound Matrix healed chronic wounds by leading the healing process to a more acute wound state [53].

### 2.3. Acellular Amniotic Membrane (AAM)

Amniotic membrane is the innermost layer of fetal placenta. ECM scaffolds derived from human amniotic membranes, termed AMM, have been commercialized for skin wound healing, such as the SURFFIXX^®^, AmnioBand^®^, Biovance^®^, and EpiFix^®^ [21,54]. Featuring anti-bacterial, immunomodulatory, and pain-reducing properties, AAM can significantly promote the healing of various kinds of cutaneous wounds, such as superficial or partial thickness burns, pressure sores, and chronic leg ulcers [55,56]. According to a systematic review and meta-analysis study, the safety and efficiency of AAM have been confirmed in the treatment of split-thickness skin graft donor sites [57]. For chronic diabetic foot ulcers, a shorter time to complete healing and a higher proportion of complete healing were observed in the AAM group when compared with the standard wound care group [58].

### 2.4. Other ECM Membranes

Beyond ADM, SIS and AAM, other ECM membranes have been investigated for skin wound healing, such as decellularized membranes derived from mesothelium and forestomach. Decellularized mesothelium membranes are scaffolds obtained from a simple squamous epithelium, which lines the walls of body cavities, such as the pleura, peritoneum, and pericardium [17,30]. In 2020, Alizadeh et al. have developed an ovine decellularized pericardium, which seems appealing for skin regeneration [17]. In another example, Endoform^®^, a forestomach-derived ECM membrane, was efficient in inhibiting a broad spectrum of matrix metalloproteinases and has been used for the healing of acute and chronic wounds [46,59].

## 3. Limitations of Traditional ECM Membranes

Although traditional ECM membranes possess excellent biological characteristics and have shown efficiency in the clinical treatment of skin wounds, there are some shortcomings hindering their broad applications. Firstly, the reagents and decellularization methods used in the preparation of scaffolds usually damage the network of natural tissues, which can lead to a rapid degradation and poor mechanical behavior of the final products [60]. Secondly, it is well-known that the accumulation of bacteria in the wounds can mediate the production of inflammatory cytokines and thus promote wound inflammation [61]. Considering that most ECM membranes do not possess antibacterial property, the risk of possible transmission of fungal, bacterial, or viral infections should be carefully addressed to avoid any unfavorable complications [62]. Furthermore, due to the heterogeneity of bio-derived materials, developing standard protocols to improve the consistency of ECM membranes is necessary for future clinical applications [60]. To conquer these limitations, various scaffold modification strategies, such as crosslinking, blending with other materials, and adding bioactive substances and/or functional particles, have been studied to improve the performance of traditional ECM scaffolds [21]. In the following sections, we will summarize current strategies to address these challenges.

## 4. Scaffold Modification for Advanced ECM-Based Membranes

### 4.1. Crosslinking

Crosslinking is a widely used scaffold modification strategy, which can greatly increase the enzymatic resistivity of ECM scaffolds and improve their biomechanical features. To prepare crosslinked ECM-based membranes, the common methods include chemical crosslinking and physical crosslinking. 

#### 4.1.1. Chemical Crosslinking

Many chemical crosslinking agents can serve as a powerful tool to crosslink the polymeric backbone of ECM scaffolds with a high crosslinking degree [63,64]. It should be noted that the feasibility of biomedical applications of some traditional crosslinking agents, such as glutaraldehyde, is still controversial because of the relatively high toxicity and the unfavorable results after implantation, especially the severe immune rejection of scaffolds, the reduced hemocompatibility, and tissue calcification [60,65,66,67,68]. In that case, naturally occurring small molecule agents, which possess lower toxicity, have been explored for the potential applications for ECM scaffold modification [11,69]. Particularly, some of these naturally occurring crosslinkers have attractive characteristics for broad biomedical applications, such as the abilities to alleviate inflammatory responses and to inhibit the initiation of tissue calcification [65,70,71].

For traditional ECM membranes, the applications of some natural crosslinking agents, such as genipin, quercetin, and proanthocyanins, have been investigated in recent years (Table 1) [65,72,73]. Gobinathan et al. reported that genipin-crosslinked AAM possessed a slower degradation rate and a lower swelling percentage than those of the AAM alone [73]. In addition, the genipin-crosslinked ADM showed significant reductions of amino acids, resulting in 37.75% in lysine, 22.89% in arginine, 21.88% in asparagine, 28.81% in glycine, and 19.48% in alanine [72]. Comparing with genipin-crosslinked ADM, quercetin-crosslinked ADM was more suitable for the applications where a lower crosslinking degree and a lower scaffold stiffness were required [72]. In vitro assays of proanthocyanins-crosslinked SIS showed that the crosslinking procedure resulted in an improved mechanical property and better anti-calcification potential [65]. 

Interestingly, some natural macromolecules can serve as crosslinking agents after proper modifications. As the modified products of chitosan, some chitosan derivatives preserve the antibacterial property, hemostatic and analgesic effects of chitosan, and importantly they acquire several unique properties such as excellent solubility and pH sensitivity (Table 1) [74]. These properties make them promising candidates for crosslinking wound healing biomaterials. For instance, after crosslinking ADM with oxidized 2-hydroxypropyl trimethylammonium chloride chitosan (OHTCC), the obtained scaffolds possessed better physicochemical characteristics, including improved tensile strength, enhanced antibacterial activity, and better enzymatic stability [75]. To further avoid the possible cytotoxicity of OHTCC, Zheng et al. have synthesized epoxidized N-(2-hydroxypropyl)-3-trimethylammonium chitosan chloride (EHTCC) and successfully produced an EHTCC-crosslinked ADM, which showed not only improved mechanical properties, thermal stability, and hydrophilicity but also excellent cellular compatibility and wound healing capacity [76].

**Table 1:** Recent studies about crosslinked ECM-based membranes.

Materials	Crosslinking Methods	Physical and Chemical Properties	Biological Results	Ref.
GP-AAM	GP crosslinking	Lower swelling ratio	Improved anti-collagenase degradation ability	[73]
Quercetin-ADM	Quercetin crosslinking	Improved mechanical strength and stiffness; Reorientation of the amino groups	/	[72]
PC-SIS	PC crosslinking	Improved max load and elastic modulus; Improved anti-calcification property	Improved anti-collagenase degradation ability; Facilitated cells organization, enhanced ECM deposition, and promoted functional gene expression	[65]
OHTCC-ADM	OHTCC crosslinking	Improved thermal stability; Improved tensile strength	Improved anti-collagenase degradation ability; Improved antibacterial activity; Preserved good cytocompatibility	[75]
EHTCC- ADM	EHTCC crosslinking	Improved mechanical properties; Improved thermal stability; Improved hydrophilicity	Excellent cellular compatibility and wound healing capacity	[76]
Riboflavin/UV-AAM	UV crosslinking with riboflavin as photosensitizer	Improved young’s modulus and ultimate tensile strength; Decreased water content	Improved anti-collagenase degradation ability; Preserved good cytocompatibility	[77]

ECM: extracellular matrix; GP: genipin; AAM: acellular amniotic membrane; ADM: acellular dermal matrix; PC: procyanidins; SIS: small intestinal submucosa; OHTCC: oxidized 2-hydroxypropyltrimethyl ammonium chloride chitosan; EHTCC: epoxidized N-(2-hydroxypropyl)-3-trimethylammonium chitosan chloride.

#### 4.1.2. Physical Crosslinking

Physical crosslinking provides a safe and simple method for introducing new crosslinks within the acellular matrices. Ultra-Violet (UV) irradiation, which can crosslink and sterilize materials simultaneously without the introduction of exogenous toxic chemicals, is a commonly employed physical crosslinking technique for ECM-based membranes. Importantly, UV irradiation often forms bonds between the aromatic residues of polypeptide chains rather than the basic and acidic side chains, which may affect cell behaviors [64,78]. However, due to the unsatisfied crosslinking efficiency of UV irradiation, this technique is usually utilized as an adjunctive method. To increase the crosslinking efficiency, researchers often combine UV irradiation with photosensitizers, such as the addition of riboflavin (Table 1) [79,80]. In an interesting study by Arrizabalaga et al., riboflavin/UV-crosslinked AAM showed superior anti-biodegradation behavior, and maintained the ability to support the proliferation and differentiation of adipose-derived stem cells [77]. Meanwhile, the mechanical properties of riboflavin/UV-crosslinked membrane were about triple that of the noncrosslinked AAM [77].

### 4.2. Combining ECM with Other Biomaterials

In addition to the high process flexibility and fine biocompatibility, synthetic biomaterials are advantageous in controlling the degradation and mechanical properties of scaffolds [81,82]. Various synthetic polymers, such as polyurethane, polyvinyl alcohol, and polycaprolactone, have been used to fabricate composite scaffolds to meet the need of physicochemical properties for skin wound healing [83,84,85,86,87]. For example, trough the assembling of an AAM sheet with a polycaprolactone nanofiber mesh, it was reported that a four- to ten-fold improvement in the suture retention strength, toughness, and ultimate tensile strength can be achieved in the bilayer membrane [84]. 

Besides synthetic polymers, bio-derived materials have been utilized as additives to enhance the performance of traditional ECM membranes [88,89,90,91]. In 2019, Dhasmanathe et al. fabricated a series of composite silk fibroin (SF)/ADM membranes through the dip-coating of ADM in SF solutions at concentrations of 5%, 10%, and 15%, respectively [92]. Among them, ADM modified with 5% SF protein, whose porosity and pore size were closest to ADM, showed faster wound contraction, better re-epithelization, and scarless healing in the full-thickness skin wounds as compared to the pure ADM group [92]. In another study, Yang et al. successfully developed a novel chitosan/AAM bilayer membrane by using the freeze-casting method; importantly, the bilayer membrane possessed good wound healing ability for diabetic wounds [88]. Interestingly, it has been observed that blood components can promote the activity of traditional ECM membranes. Activated by platelet rich plasma and calcium chloride composition, AAM showed better wound healing outcomes in a mouse skin wound model, which was evidenced by better regeneration of epidermis, hair follicles and basement membrane [90].

### 4.3. Loading ECM with Therapeutic Agents

Normal skin wound healing involves multiple pathways to go through the phases of hemostasis/inflammation, proliferation, and remodeling [10]. Bacterial pathogens can reach the wound sites and produce endotoxins at the wound beds [93]. The endotoxins can stimulate the secretion of proinflammatory cytokines, which in turn decrease the syntheses of growth factors and collagens that are favorable for wound healing, and finally result in critical colonization and invasive infection of pathogens [94]. Therefore, in cases of ECM membranes with little antibacterial property, wound infection and inflammation are crucial issues that need to be well addressed. 

#### 4.3.1. Loading with Antibacterial Agents

To enhance the antibacterial property of ECM-based wound dressings, different antibacterial drugs, including rifampicin, ciprofloxacin, gentamycin, and minocycline, have been used as additives (Table 2) [13,95,96,97]. Gentamicin loaded SIS, for instance, was proved to exert sufficient antimicrobial activity against a broad array of pathogens (*E. coli*, *S. epidermidis*, methicillin-resistant *S. aureus*, *P. aeruginosa*, *S. marcescens*, and *S. aureus*), and particularly, the antibacterial effects could be maintained for up to 7 days [98]. Similarly, Goller et al. have loaded the chitosan (CS)-porcine urinary bladder (UBM) wound dressings with minocycline or rifampicin [99], which resulted in antimicrobial activities against *E. coli* and *S. aureus*. The drug release rate and the antibacterial effect of these scaffolds could be adjusted by regulating the ratio of CS and UBM [99].

Further, antibacterial nanoparticles with high surface area-to-volume ratios have also been developed as popular additives [109,110,111]. The unique physiochemical properties of nanoparticles facilitate them to penetrate the skin tissue and inhibit the growth of bacterial through multiple mechanisms such as the blocking of cellular respiration, the disrupting of bacterial membranes, and the condensing of bacterial DNA [100,112]. In particular, the antibacterial efficacy of nanoparticles can be modulated by changing their micromorphology, zeta potential, surface functionalization, and hydrolytic stability [112,113]. 

Currently, various kinds of antibacterial metal nanoparticles, like silver nanoparticles, zinc oxide nanoparticles, and titanium oxide nanoparticles, have been incorporated into membranous ECM scaffolds to produce advanced wound dressings (Table 2) [100,101,102,114,115]. Among them, silver nanoparticles have been widely used in the treatment of acute and chronic wounds. Through the immersion of scaffolds in silver nanoparticle suspensions at concentrations of 0% to 1%, ADM have been used to fabricate nanoparticle-loaded ADM [100]. These silver nanoparticle-functionalized ADMs were without significant cytotoxicity, but showed a concentration dependent suppression of the growth of *Pseudomonas aeruginosa* and *Staphylococcus aureus* [100]. Furthermore, by using a similar scaffolding method, silver nanoparticles have been loaded onto SIS membranes, and the modified scaffolds were effective in the treatment of *Pseudomonas aeruginosa*-infected burn wounds [101]. In the wounds treated with silver nanoparticles loaded SIS membranes, the expression levels of IL-6 and C-reactive protein were significantly lower than that of the pure SIS group, accompanying with less inflammation, more re-epithelization, and better neovascularization [101]. Zinc oxide nanoparticles, another frequently used agent to avoid wound infection, are compatible with biological system, and their biocompatibility and antimicrobial activity are related to the particle size [116]. After loading with zinc oxide nanoparticles, it was observed that AAM wound dressings showed a dose-dependent antibacterial activity of Gram-positive (*S. mutans*, *S. aureus*, *L. fusiformis*, and *E. faecalis*) and Gram-negative bacteria (*P. vulgaris*, *S. sonnei*, *C. freundii*, and *P. aeruginosa*) [102].

In addition to drugs and nanoparticles, antiseptic peptides have been utilized to generate antibacterial biomaterials for wound healing. For instance, Kasetty et al. showed that, after the addition of thrombin-derived host defense peptides, the modified ADM scaffolds exert antibacterial activity against *E. coli*, *P. aeruginosa*, and *S. aureus* [103]. Furthermore, these peptides can protect ADM from bacteria-mediated degradation and endow the biomaterial with endotoxin-blocking property [103].

#### 4.3.2. Loading with Anti-Inflammatory Agents

Because prolonged inflammation may impair normal wound healing, many anti-inflammatory substances, such as drugs and nucleic acids, have been loaded on wound dressings to accelerate wound healing. For example, dexamethasone (Dex) and silver sulfadiazine (AgS) have been used to strengthen the anti-inflammatory function of SIS (Table 2) [13]. With the release of Dex and AgS, the SIS scaffolds effectively suppressed macrophage infiltration [13]. 

To accelerate chronic wound healing, it is feasible to introduce therapeutic nucleic acids into scaffolds to inhibit or support the expression of specific proteins [117]. ECM scaffolds loaded with anti-inflammation nucleic acids are promising scaffolds for wound healing [117]. For anti-inflammatory purpose, miRNA-223 5p mimic has been used as an additive to control the polarization of macrophages into a M2 phenotype [118]. Other therapeutic nucleic acids like TNF-α siRNA and miR-146a can also endow skin wound healing scaffolds with anti-inflammatory properties [119].

#### 4.3.3. Loading with Antioxidant Agents

At the wound bed, the phagocytizing of microorganisms or foreign debris by inflammatory cells may lead to the generation of high concentrations of reactive oxygen species (ROS) [120]. Excessive ROS can induce oxidative stress, which can result in a series of unfavorable effects including deferred cellular behaviors, extended inflammation period, reduced re-epithelization, and diminished angiogenesis. Thus, excessive oxidative stress is detrimental to normal wound healing [105,121,122]. 

Antioxidant agents, such as ferulic acid, alpha-lipoic acid, and Keap1 siRNA, are capable of eliminating free radicals and have been successfully loaded in different nanoparticulate delivery systems to treat skin wounds [104,105,119]. For example, to strengthen the antioxidant ability of ADM scaffolds, Pesaraklou et al. have immersed the ADM scaffolds in a cerium oxide nanoparticle (CeO_2_ NP) solution to produce a CeO_2_ NP-ADM scaffold (Table 2) [104]. Compared to ADM alone, the CeO_2_ NP-ADM scaffold showed improved free radical scavenging ability, enhanced cell survival rate, better collagen content, and higher tensile strength [104]. Besides CeO_2_ NP, carbon nanodots (CN) present another novel antioxidant agent that can accelerate skin wound healing [123]. In 2020, Bankoti et al. have modified CS-ADM with CN to achieve a good ROS scavenging property (Table 2) [105]. After 21 days of treatment, diabetic wounds covered with CN-CS-ADM scaffolds, in which human amniotic membrane derived stem cells were also loaded, showed rapid wound closure, complete reepithelialization, and distinct formation of organized dermal epidermal junctions, suggesting that it may serve as a promising therapeutic strategy for chronic wounds [105]. 

#### 4.3.4. Loading with Other Therapeutic Agents

Growth factors, which are capable of mediating tissue repair through the interaction with specific cell surface receptors, play a vital role in the acceleration of chronic wound healing [124]. Several growth factors, such as fibroblast growth factor, epidermal growth factor (EGF), VEGF, platelet-derived growth factor (PDGF), and TGF-β, have been introduced to skin wound healing as therapeutic agents [125,126,127]. For instance, Su et al. reported an EGF loaded hyaluronic acid (HA)-decellularized peritoneum (DP) scaffold, in which a sustained release of EGF was observed (Table 2) [106]. The addition of EGF was proved to be efficacious in the treatment of skin wounds, which resulted in a raised healing rate, promoted regeneration of skin appendages, and thicker layers of epidermis and dermis [106]. Similarly, another study showed that the healing rate and relative expressions of a-SMA and lumican were accelerated and increased after loading ADM with PDGF [128]. 

In addition to growth factors, other bioderived natural compounds, such as curcumin, tea tree oil, and honey, are promising therapeutics, because they have manifold functions in skin wound healing [108,129,130]. Curcumin, for example, is a natural polyphenolic phytoconstituent extracted from turmeric. It is favorable for skin wound healing because of its antioxidant, antimicrobial, anti-inflammatory, and antimutagenic characteristics [131,132]. After the incorporation of curcumin, modified SIS membranes were found to acquire good antibacterial ability and free radical scavenging capability, thus making them potent to neutralize the problems of oxidative stress and biofilm formation in skin wounds (Table 2) [107]. As a carbohydrate-rich natural component, honey possesses the advantages of anti-inflammatory, antibacterial, and wound healing promoting properties [133]. After its incorporation with ADM, the antimicrobial activity of honey-ADM is significantly enhanced, with a maximum bacterial growth inhibition up to 60 h for *E. coli*, and 30 h for *S. aureus* (Table 2) [108]. In addition, results of in vivo wound healing test demonstrated that the honey-ADM treated skin wounds showed controlled immune response and better wound healing results as compared to that of the ADM alone [108]. 

## 5. Fabrication Technologies for Advanced Membranous ECM-Based Scaffolds

With the development of scaffold fabrication technologies, particularly the electrospinning methods and the three-dimensional (3D) bioprinting approaches, advanced ECM-based membranes with improved performance and/or tunable properties have emerged in the area of skin wound healing [45,46,47,48,49,50,51].

### 5.1. Electrospinning

**Figure 2:**
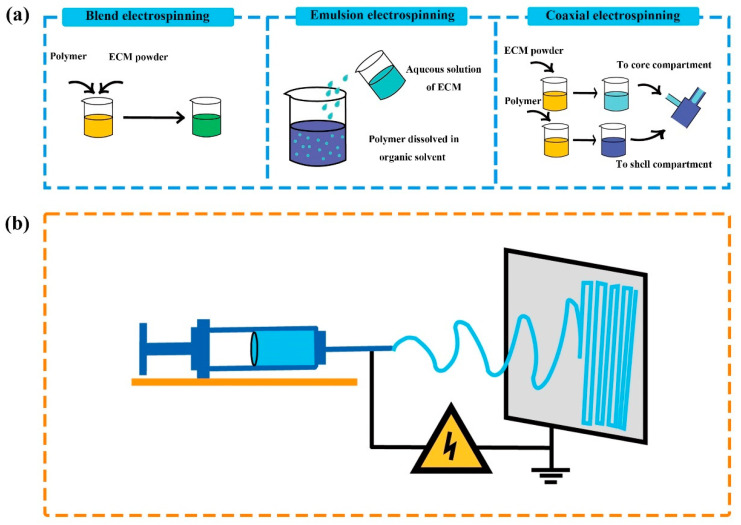
Schematic diagram of electrospinning technology for the preparation of membranous ECM-based scaffolds. (**a**) Different methods have been developed for the preparation of ECM-based bioinks before electrospinning; (**b**) The fabrication of ECM-based membranes by using an electrospinning device. ECM: extracellular matrix.

Electrospinning is a versatile and relatively economic technique, which enables easy fabrication of fibrous mats [134]. An electrospinning device usually comprises high voltage or low voltage supplies, syringe pumps, spinnerets, and collectors [135,136]. During the operation of the devices, the potential difference established between the spinneret and the collector facilitates the formation and deposition of fibers with controllable diameters ranging from nano-size to micro-size [134,135]. In the literature, electrospun ECM-based membranes have been reported by electrospinning the ECM raw materials with organic solvents and/or macromolecules such as gelatin, silk fibroin, poly hydroxyalkanoate, polycaprolactone, and polylactic acid [136,137,138,139,140,141,142]. Based on the requirement of scaffolds, various electrospinning approaches, including the emulsion electrospinning, blend electrospinning, coaxial electrospinning, simultaneous electrospin-electrospraying, and post spinning modifications, are optional for the fabrication of scaffolds (Figure 2) [139,143,144,145,146,147,148].

With high surface-to-volume ratios, electrospun fibers are capable of recruiting repair cells and have deep interactions with the wound area [149,150,151,152]. They can be designed for specific purposes, such as to improve the mechanical strength of scaffolds, to enhance the anti-degradation ability, to carry therapeutic agents, and for gene therapy applications [13,153,154]. For example, by using the method of blend electrospinning, Kim et al. have developed an electrospun poly(l-lactide-co-caprolactone) (PLCL)/ECM wound dressing, whose tensile stress (2.23 ± 0.44 MPa) was similar to that of the PLCL group, but the E-modulus (2.04 ± 0.34 MPa) was significantly greater than the PLCL group (0.19 ± 0.01 MPa; *p* < 0.01) [155]. Notably, when compared with the PLCL scaffolds, the PLCL/ECM wound dressing can significantly enhance wound angiogenesis, regenerate tissues, and reduce scarring [155]. In another study, poly(ε-caprolactone-ran-L-lactide) (PCLA) was used to improve the electrospinning performance of SIS [13]. By adjusting the ratio between SIS and PCLA, scaffolds with suitable micro morphology and tensile strengths were successfully developed [13]. After loading with anti-inflammatory drugs, the scaffolds were proved to sustain a drug release period over the in vivo implantation and successfully suppressed macrophage infiltration [13]. In addition to anti-inflammation drugs, other bioactive molecules such as substance P (SP), a 11-amino-acid-long neuropeptide, can be added in the SIS/PCLA system for better wound healing performance. Compared with the PCLA and SIS/PCLA groups, SP-loaded SIS/PCLA showed more blood vessel formation, more epidermal regeneration, higher collagen density, and fewer macrophage infiltration in the wounds [91].

### 5.2. Three-Dimensional (3D) Bioprinting

Three-dimensional (3D) bioprinting is a versatile technique to produce design-driven scaffolds [156,157,158]. After the loading of medical image data or specific software, bioinks can be deposited in the correct coordinates to create a 3D structure based on a predefined spatial model [159,160]. The ECM solutions with suitable concentrations and rheological properties can be utilized as bioinks for 3D bioprinting [161]. Generally, the solubilization of raw ECM materials can be achieved by a series of operations, such as pulverization, enzymatic digestion, and neutralization [162,163]. However, because of the fragility and poor printability of ECM-based bioinks, the bioprinting process is very challenging [161]. To tackle this obstacle, several strategies have been developed. For instance, through the addition of photo initiator at proper concentrations, a novel SIS-based photocrosslinkable bioink has been manufactured [164]. Besides photo initiator, the performance of ECM-based bioinks can be modulated by other biomaterials [157]. Kim et al. reported that the printability and mechanical properties of ECM-based bioink could be enhanced by loading the ECM powder with a mixture of gelatin, hyaluronic acid and fibrinogen [165]. 

**Figure 3:**
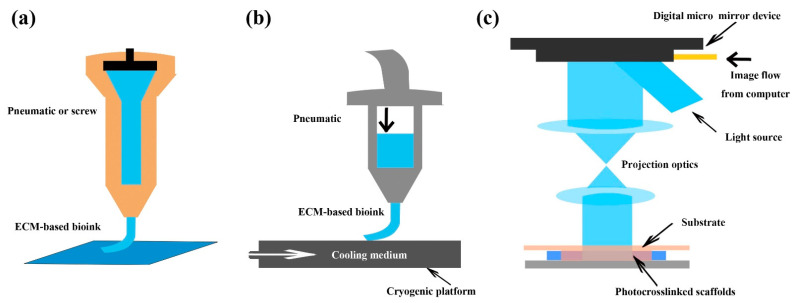
Three-dimensional (3D) printing technology for the preparation of membranous ECM-based scaffolds. (**a**) Traditional extrusion bioprinting; (**b**) Cryogenic free-form extrusion bioprinting; (**c**) Digital light processing bioprinting. ECM: extracellular matrix.

Among various kinds of 3D printing techniques, two approaches are commonly used for the printing of ECM-based bioinks, namely the extrusion-based printing method and the digital light processing (DLP) printing method [157,162,166,167]. In the extrusion-based printing technique, ECM-based bioinks are extruded through a needle-syringe-type system and deposited at the pre-defined spatial locations through an automated robotic system (Figure 3) [168,169]. With excellent printing accuracy, efficiency, and working conditions, DLP printing is an attractive technology to fabricate photosensitive scaffolds. During the process of DLP printing, the photocrosslinkable bioinks are crosslinked by a projection light generated by the optical micro-electromechanical technology, and finally form a stable structure with proper mechanical properties (Figure 3) [166,170]. In addition to the SIS-based photocrosslinkable bioink, other types of photocrosslinkable bioinks have been reported, mainly through the addition of a mixed solution containing gelatin methacrylate hydrogel and photoinitiator [166].

To print SIS ink, the employment of a specialized free-form extrusion bioprinting system, which is composed of a 3D robot platform, a pneumatic dispensing system, and a cryogenic stage (Figure 3), have been reported [171]. After crosslinking with 1-ethyl-3-(3-dimethylaminopropyl) carbodiimide, the fabricated scaffolds showed a porous microstructure and possessed adequate strength for cell growth during skin wound repair [171]. In addition, full thickness 3D human skin composed of cells and skin-derived ECM could be established through a combination of the extrusion-based printing technology and the inkjet-based printing technology [169]. 

In addition, to fabricate skin grafts with structure closer to natural skin, a perfusable/vascularized skin equivalent composed of epidermis, dermis, and hypodermis has been created by printing multiple bioinks through an in-house-built hybrid printing system [172]. In another study, a 3D printed multilayer skin has been produced, in which human skin keratinocyte loaded gelatin methacrylamide served as the epidermal layer, fibroblasts loaded ADM as the dermis, and human umbilical vein endothelial cells loaded gelatin methacrylamide as the vascular network and framework [173]. In vivo results showed that the printed multilayer skin equivalent accelerated wound healing, as seen by improved re-epithelization, dermal ECM secretion, and angiogenesis [173].

## 6. Future Perspectives

Although the use of traditional ECM membranes for skin wound healing can go back decades, new types of decellularized membranes, such as those produced from forestomach, chorion, pleura, and peritoneum, are emerging [20,59,174]. The development of new convenient cell extraction strategies, such as the use of supercritical carbon dioxide for scaffold decellularization, has been reported in the literature [175,176,177,178]. This progress offers more technical options and raw ECM materials for the development of new wound dressings. Particularly, advances in chemistry, composite materials, nanotechnology, and process technology enable the production of advanced membranous ECM-based scaffolds.

However, there are many “bottle neck” problems that need to be tackled. For instance, in large skin wounds, to achieve efficient tissue regeneration and functional recovery, it is necessary to build scaffolds with precise tissue details of natural skin, especially the hair follicles and sweat glands. However, this remains a big technical challenge. Future research should put a strong emphasis on the development of appendage-bearing scaffolds, in which the specific macromolecular components and the cells of skin appendages are arranged in a predefined architecture. With the emergence of 4D printing and the development of the handheld skin printer, these apparatuses may bring a revolutionary breakthrough to facilitate the rapid production of a desired ECM-mimicking scaffold with larger areas, more exquisite structures, and diversified functions [10,162,179].

It is well known that many therapeutic agents work well in the wound healing stages. At the early stage of wound healing, anti-inflammatory agents and coagulation factors are required, whereas growth factors are required in the proliferation and ECM remodeling stages [180]. The development of ECM-based scaffolds that can release particular therapeutic agents to meet the need of different stages of wound healing will contribute a lot to improving the wound healing outcome [180]. Besides therapeutic agents, stem cells, such as pluripotent stem cells, have shown inspiring results for skin regeneration, especially the newborn of pigmented hair follicles and sebaceous glands [181]. Consequently, the development of stem cells/ECM-mimicking scaffold constructs would provide a viable option for patients who failed in wound healing. Furthermore, considering the fact that many additives and solvents, which are a must for use in the scaffold modification process, are usually toxic or with unclear metabolic mechanisms [182], it is necessary to devote extensive efforts to solving the problem of toxic reagent residues and to search for non-poisonous substitutes [182,183,184]. 

## 7. Conclusions

After substantial efforts were devoted to scaffold modification, significant advances in the improvement and functionalization of ECM-based membranes have been made in recent years, mainly through the methods of scaffold crosslinking, blending with other biomaterials, and adding bioactive substances. Some advanced scaffold fabrication technologies have been introduced to fabricate multifunctional ECM-based scaffolds. Particularly, electrospinning and 3D printing are applicable to generating ECM-based scaffolds with predefined compositions and topography. Although the research progress is marvelous, engineering fully functional skin constructs remains a significant challenge. Future studies are necessary to detail the toxicity of advanced ECM-based scaffolds, their metabolic mechanisms, and their potential clinical applications.

## Figures and Tables

**Table 2:** Various therapeutic agents contained ECM scaffolds and their biological characteristics.

Materials	Developing Methods	Biological Characteristics	Ref.
Gentamicin-SIS	Hydrated SIS in a 40 mg/mL gentamicin solution for 2 min	Anti-*E. coli*; Anti-*S. epidermidis*; Anti-methicillin-resistant *S. aureus*, Anti-*P. aeruginosa*; Anti-*S. marcescens*; Anti-*S. aureus*	[98]
Antibiotic-CS-UBM	Dissolved the antibiotic powder (3 mg minocycline/dish or 0.5 mg rifampicin/dish) to the CS: UBM slurry in a 60 mm petri dish	Anti-*E. coli*; Anti-*S. aureus*; Adjustable drug release rates and antibacterial effects	[99]
Silver NP-ADM	Immersed ADM into a silver NP suspension at concentrations of 0 to 1% for 1 min	Anti-*P. aeruginosa*; Anti-*S. aureus*; No significant cytotoxicity	[100]
Silver NP-SIS	Immersed SIS into a 50 mg/mL silver NP suspension for 24 h	Anti-*P. aeruginosa*; Lower expression levels of IL-6 and C-reactive protein, less inflammation, more re-epithelization, and better neovascularization in the wounds of the silver NP-SIS group than that of the pure SIS group.	[101]
ZnO NP-AAM	Immersed AAM into a 75 µg/mL ZnO NP suspension for 3 h	Anti-Gram-positive bacteria (*S. aureus*, *S. mutans*, *E. faecalis*, and *L. fusiformis*); Anti-Gram-negative bacteria (*S. sonnei*, *P. aeruginosa*, *P. vulgaris*, and *C. freundii*)	[102]
THDP-ADM	Coated ADM with a 10 mL THDP solution at concentrations of 0.647, 1.62 and 3.24 mM	Anti-Gram-positive bacteria (*S. aureus*); Anti-Gram-negative bacteria (*E. coli*, *P. aeruginosa*); Endotoxin-blocking property	[103]
Dex-SIS AgS-SIS	Electrospun solutions containing Dex-SIS or AgS-SIS	Suppressed macrophage infiltration	[13]
CeO_2_ NP-ADM	Immersed ADM into a CeO_2_ NP suspension at concentrations of 1 to 20 mg/mL for 24 h	Antioxidant property	[104]
CN-CS-ADM	Added CN to the CS-ADM at a concentration of 1.5 mg/mL	Good ROS scavenging property	[105]
EGF-HA-DP	Immersed HA-DP into a 1 μg/mL EGF solution for 12 h	Raised wound healing rate; Promoted regeneration of skin appendages; The regeneration of thicker epidermis and dermis layers	[106]
Curcumin-SIS	Added SIS to the curcumin solutions at concentrations of 0.1, 0.5 and 1% for 30 min	Anti-*E. coli*; Anti-*S. aureus*; Free radical scavenging capability	[107]
Honey-ADM	Immersed ADM into the honey solutions at concentrations of 5%, 10%, 15% for 30 min	Anti-*E. coli*; Anti-*S. aureus*; Controlled immune response	[108]

ECM: extracellular matrix; SIS: small intestinal submucosa; CS: chitosan; UBM: porcine urinary bladders; ADM: acellular dermal matrix; NP: nanoparticle; ZnO: Zinc oxide; AAM: acellular amniotic membrane; THDP: thrombin-derived host defense peptides; Dex: dexamethasone; AgS: silver sulfadiazine; CeO2: cerium oxide nanoparticles; CN: carbon nanodots; EGF: epidermal growth factor; DP: decellularized peritoneum.
